# Implications of Agile Values in Software Engineering for Agility in Breast Cancer Treatment: Protocol for a Comparative Study

**DOI:** 10.2196/53124

**Published:** 2023-12-05

**Authors:** Yousra Odeh, Mahmoud Al-Balas

**Affiliations:** 1 Software Engineering Department Faculty of Information Technology Philadelphia University Amman Jordan; 2 Department of General Surgery, Anesthesia and Urology Faculty of Medicine Hashemite University Zarqa Jordan

**Keywords:** agile breast cancer treatment, breast cancer, breast cancer treatment, agile, software engineering, agile software engineering, oncology, agile values, multidisciplinary research, agility in health care, agile oncology practice

## Abstract

**Background:**

Breast cancer treatment has been described as a dynamic and patient-centered approach that emphasizes adaptability and flexibility throughout the treatment process. Breast cancer is complex, with varying subtypes and stages, making it important to tailor treatment plans to each patient’s unique circumstances. Breast cancer treatment delivery relies on a multidisciplinary team of health care professionals who collaborate to provide personalized care and quick adaptation to changing conditions to optimize outcomes while minimizing side effects and maintaining the patient’s quality of life. However, agility in breast cancer treatment has not been defined according to common agile values and described in language comprehensible to breast cancer professionals. In the rapidly evolving landscape of breast cancer treatment, the incorporation of agile values from software engineering promises to enhance patient care.

**Objective:**

Our objective is to propose agile values for breast cancer treatment adopted and adapted from software engineering. We also aim to validate how these values conform to the concept of agility in the breast cancer context through referencing past work.

**Methods:**

We applied a structured research methodology to identify and validate 4 agile values for breast cancer treatment. In the elicitation phase, through 2 interviews, we identified 4 agile values and described them in language that resonates with breast cancer treatment professionals. The values were then validated by a domain expert and discussed in the context of supporting work from the literature. Final validation entailed a domain expert conducting a walkthrough of the 4 identified agile values to adjust them as per the reported literature.

**Results:**

Four agile values were identified for breast cancer treatment, and among them, we validated 3 that conformed to the concept of agility. The fourth value, documentation and the quality of documentation, is vital for breast cancer treatment planning and management. This does not conform to agility. However, its nonagility is vital for the agility of the other values. None of the identified agile values were validated as partially conforming to the concept of agility.

**Conclusions:**

This work makes a novel contribution to knowledge in identifying the first set of agile values in breast cancer treatment through multidisciplinary research. Three of these values were evaluated as conforming to the concept of agility, and although 1 value did not meet the concept of agility, it enhanced the agility of the other values. It is anticipated that these 4 agile values can drive oncology practice, strategies, policies, protocols, and procedures to enhance delivery of care. Moreover, the identified values contribute to identifying quality assurance and control practices to assess the concept of agility in oncology practice and breast cancer treatment and adjust corresponding actions. We conclude that breast cancer treatment agile values are not limited to 4.

**International Registered Report Identifier (IRRID):**

RR1-10.2196/53124

## Introduction

### Background

Breast cancer is the most frequent cancer diagnosis and the main cause of cancer mortality in women globally [1]. Through the 2 past decades, traditional breast cancer treatment (BCT) has been the standard of care and has led to significant improvements in patient outcomes [2]. However, the lack of consideration of the unique characteristics of each patient’s cancer can lead to ineffective and nontargeted therapy with adverse and toxic side effects, reduced quality of life, and, in some cases, a resulting delay in detecting progression [2-4]. Thus, today, BCT is shifting toward more personalized approaches that enhance the patient experience while considering their diverse characteristics and evolving conditions [3,5-7].

Different treatment strategies may be required for each subtype of breast cancer, and genetic variation within tumors may complicate aspects of treatment. Developing tailored medicines that target certain genetic alterations or molecular subtypes is one of the main challenges in BCT [8]. Treatment planning has become more difficult as a result of the increased emphasis on personalized medicine, which calls for adjusting plans depending on a patient’s genetic profile [8]. Optimizing therapeutic results and reducing the risk of treatment resistance require customizing therapies to fit the genetic features of each patient’s tumor [9]. A patient’s values and preferences must be taken into account in addition to clinical factors while deciding on the best course of action [10].

Further complication arises from managing treatment side effects and maintaining a patient’s quality of life, particularly with regard to the level of depression both before and after treatment, as highlighted in the work of Salibasic and Delibegovic [11] and Breidenbach et al [12]. It can be difficult to strike a balance between a treatment’s effectiveness and possible effects on the patient’s well-being. Additionally, there are obstacles associated with the timing and order of various treatment modalities, including radiation therapy, hormone therapy, immunotherapy, chemotherapy, and surgery. A multidisciplinary approach to care is necessary to determine the most effective sequence and combination of therapies, which necessitates a comprehensive evaluation of the tumor characteristics, patient’s overall health, and adverse side effects [13].

In the literature, all these reported treatment challenges, efforts, and strategies lack consideration and identification of their respective agility values. According to the *Oxford Dictionary*, the word *agility* means “the ability to move quickly and easily” [14]. *Value* means “the quality of being useful or important” or “beliefs about what is right and wrong and what is important in life” as a meaning related to *principle* or *standard* [15]. Thus, to improve BCT approaches and strategies and to overcome these challenges, it should be assessed whether they meet agility values and to what extent. This calls for identifying standards for the ability to move quickly, easily, and effectively in this complex disease, that is, defining *agility values* for BCT. Although the literature emphasizes addressing some agility values, such as patient-centric processes, personalization, and a multidisciplinary approach to decision-making and treatment plan design, there is still no validated definition of agility values for BCT. The complexity of BCT, shaped by factors ranging from tumor characteristics to patient preferences, highlights the need to identify common agile values to help in overcoming the above challenges. By identifying and promoting these values, health care organizations can create an agile culture of interactions, continuous learning, and improvement.

Multidisciplinary research has inspired individuals and communities, offered knowledge returns to individuals, and improved environment outcomes. In order to improve BCT and research, the different domains of software engineering and BCT may complement each other in a number of ways. Recent software development projects are adopting the agile approach to produce high-quality, useful software, in contrast to the traditional approach, which has limited flexibility, low user involvement, a long and delayed time-to-market cycle, late detection of errors, and high cost [16,17]

The concept of agility has been successfully described in the software engineering field over decades [17]. In software engineering, agility is a mindset, and its 4 values are not solid rules to follow [18]. In this context, it is possible to use the 4 agile values of software engineering for providing a shared framework for BCT teams to work together effectively and deliver value to their stakeholders while adapting to changing circumstances in complex environments and improving collaboration for better decision-making through a human-centric approach. Agile values emphasize the importance of individuals and interactions. This human-centered approach helps in building collaborative and motivated teams. The 4 agile values in software engineering are “individuals and interactions over processes and tools,” “working software over comprehensive documentation,” “customer collaboration over contract negotiation,” and “responding to change over following a plan” [18]. The agile approach has enhanced value delivery to customers when user-centered experience and design are considered in the work of agile teams [19-21]. The agile approach has been used in the health care sector to translate the delivery of care from traditional methods into personalized ones, making care patient-centric, involving the patient in decision-making, enhancing continuous improvement and learning, and encouraging collaboration [22-26].

The creators of the agile values and principles have placed collaboration and people front and center, as the success or failure of a software project is highly dependent on them and not on the processes, tools, or the technology invested in it. Similarly, in the BCT process, the patient can be placed at the center of care by developing a personalized treatment plan considering the patient’s preferences, values, and circumstances and engaging their families to develop accurate and well-conceived plans [18].

### Aim of the Study

In this paper, we aim at identifying agility values in BCT to drive the BCT journey and ensure that patients receive appropriate and responsive care that considers their unique needs and preferences, ultimately leading to improving the effectiveness and efficiency of BCT strategies and approaches. The 4 identified agile values are adopted and adapted for BCT from the software engineering field. Also, we aim to investigate and validate how these values conform to the concept of agility in the breast cancer context through work reported in the literature. This should allow us to assess how they conform to the concept of agility in comparison with agile values in the field of software engineering. The reason behind adopting and adapting agility values from software engineering is that this discipline was founded to engineer effective solutions for complex problems while considering different aspects of continuous change. According to a domain expert, this is a noted similarity to the BCT discipline.

## Methods

**Figure 1:**
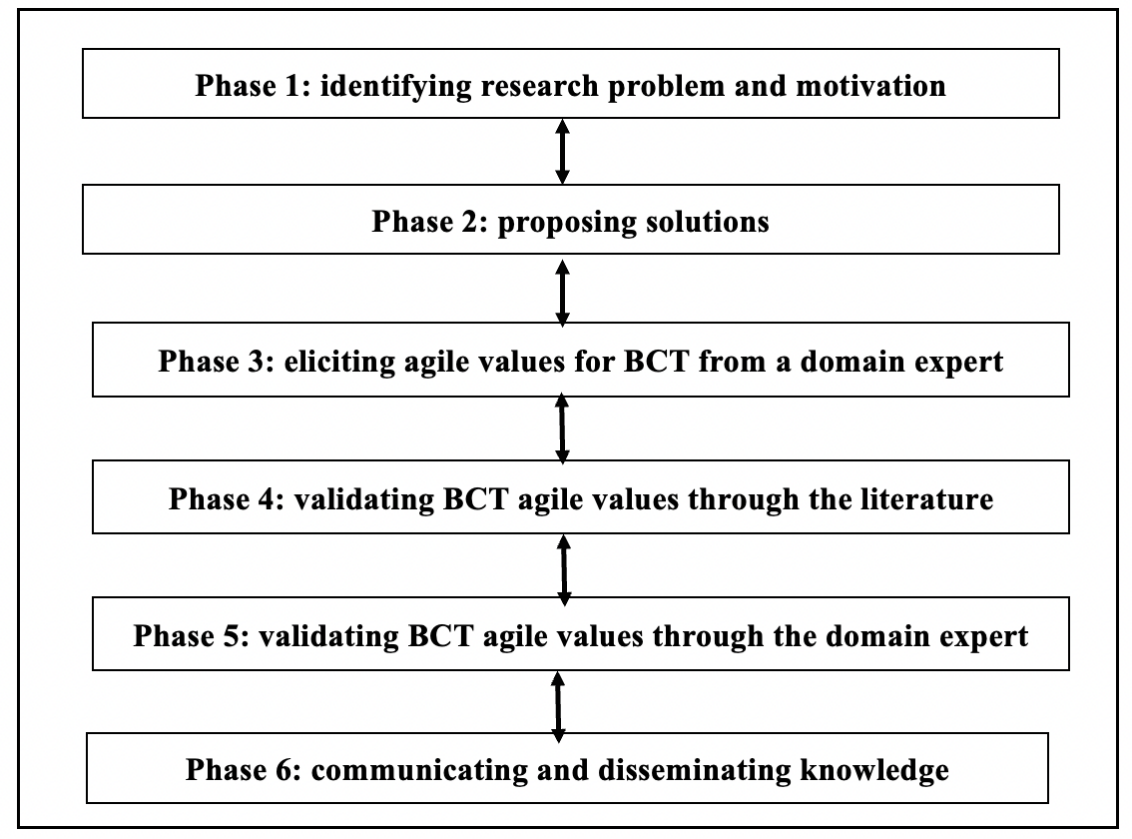
Research methodology.

Here, the applied research methodology has 6 overlapping phases (Figure 1). This encourages researchers to actively engage with the problem-solving process, resulting in outcomes that align with the identified and validated agile values for BCT; this is anticipated to have a direct impact on BCT, health care, and society. As the methodology is described as a problem-solving process, it starts by identifying the research problem and the associated motivation. This leads to identifying the desired solution, which allows identifying the corresponding BCT agile values. Then, an elicitation phase is conducted to elicit the agile values for BCT from the domain expert after reviewing and amending the software engineering agile values into language that is reasonable for BCT professionals. In the fourth phase, we review the literature to provide references with the aim of validating these values. In the fifth phase, the domain expert conducts a walkthrough of the 4 BCT agile values as a final validation before communicating them to the research community in phase 6.

### Phase 1: Identifying Research Problem and Motivation

According to a literature review, there is a gap in promoting the agility of BCT with respect to well-identified values, although the literature reports huge efforts in patient-centered and personalized BCT. It is essential for patients to have open, transparent, and personalized communication with their care team professionals and health providers about their treatment approach and whether it is agile or not. Patients are empowered when they are aware of how BCT is delivered, particularly if agile mindsets are leading the journey. This encourages them to take an active role in making decisions about their care, ask pertinent questions, and advocate for the appropriate treatment options that meet their individual needs and circumstances. Having initial BCT agile values would pave the way to more flexible and personalized protocols, polices, and procedures to move closer to deliver patient-centric care. This also motivates policymakers to specify quality assurance processes that measure and indicate the extent of compliance to agility. Overall, this motivates health care providers to improve their care outcomes and increases patient satisfaction.

### Phase 2: Proposing Solutions

To our knowledge, there is a lack of a validated definition of agile values for BCT, although BCT professionals strive to adhere to agility, as reported in the literature. The authors seek to propose and identify BCT agile values that are adopted and adapted from the software engineering field.

### Phase 3: Eliciting Agile Values for BCT From a Domain Expert

The 4 agile values of software engineering were reviewed and rearticulated into language that is tailored to BCT professionals through 2 interview elicitation sessions conducted with a BC oncologist. The outcome here was the 4 identified BCT agile values, which are discussed below in phase 4 through work referenced from the literature. The outcomes of this phase are the following:

- Value 1: Individuals and interactions over processes and tools
- Value 2: Documentation and quality of documentation is as high priority as delivering an appropriate patient-centered treatment plan
- Value 3: Patient collaboration over treatment plan design agreement
- Value 4: Responding to change over following a plan

### Phase 4: Validating Breast Cancer Treatment Agile Values Through the Literature

In this phase, we aim to reference work from literature that supports the articulation of the BCT agile values, as defined in phase 3. Below, each of the 4 BCT agile values is supported with work reported in the literature to enhance the validation.

#### Value 1: Individuals and Interactions Over Processes and Tools

In software engineering, this agile value highlights the fact that people and the communication behind developed processes and tools for achieving software development success are more important than these processes and tools themselves. Even if the team has the best, most appropriate tools, having the right people along with effective communication is still of greater importance. Regarding language for BCT professionals, this value is maintained as it is, without changing any of its wording [18,20].

In the BCT context, “working together is much more than policies, strategies, structures and processes” [27]. This can be seen in the shared decision-making (SDM) process for BCT, where the patient and everyone else is involved in delivering valuable information and reducing any mismatch between information that is delivered and the information that is needed in the complex treatment process [28]. When a patient is involved in SDM, it permits consideration of the patient’s preferences in the treatment, increasing their satisfaction and transforming the treatment process into a patient-centered one [29]. Improving patients’ engagement skills, using elicitation, performing clarification exercises for their preferences, and using formal and informal coaching are recommended to increase patients’ engagement in SDM in the BCT lifecycle. However, lack of time and resources are the biggest barriers mentioned regarding SDM [29]. This would prioritize individuals and their interactions over the tools [26,28,29]. Patient-centered communication and education, as well as coordination of care, are vital components for achieving the triple aims defined by the Institute for Healthcare Improvement that aim to improve the care experience for patient and family, improve the population’s overall health, and reduce health care capital costs [30]. According to research findings, the effectiveness of communication changes depending on the stage of the disease, and, for it to be effective, it must be adapted to the changing requirements and preferences of breast cancer patients [31,32]. Even after cancer treatment, regardless of the channel used, communications and interactions are still necessary. For example, a storytelling support group was run over the internet to break the social isolation that follows cancer [33,34].

In cancer care, the multidisciplinary team is a core central point of communication and collaboration. The National Health Service in the United Kingdom defines the multidisciplinary team (MDT) as “a group of health and care staff who are members of different organizations and professions (e.g. GPs, social workers, nurses, anesthesiologist, radiologist, pathologist, etc.,) that work together to make treatment and related services decisions of individual patients. MDTs are used in both health and care settings” [35]. In BCT planning, individuals in the MDT and their interventions can optimize care planning. For example, a nurse can stimulate interdisciplinary strategic planning that satisfies the clinical setting and improves outcomes through playing a key role in assessing the patient’s holistic needs [36]. A breast radiologist plays a vital role in the assessment and management of pain while leveraging patient communication and education, can add significant value to the care delivery process and patient care, and can improve outcomes [37]. Breast cancer patients who participated in a well-organized MDT contributed to facilitating a clear definition of their needs related to diagnosis, treatment, surveillance, and monitoring. This significantly enhanced breast cancer care planning, clarifying SDM in terms of booking appointments, referring procedures, and allocating respective roles and their responsibilities for patient care [38-40]. As a result, this MDT communication approach with the patient enhances survival rates and improves quality of life [40-44]. Although the research community reports that the MDT and patient interaction are prioritized over processes, some barriers may still hinder the effectiveness of these interactions; these are reported to include absence of leadership, individual personalities, cultural and belief systems, the need for regular clinical meetings, health care workers with double positions, availability of the workforce, specific goals of care, implementation in national health insurance, hospital bureaucracy, issues with hospital infrastructure, patients themselves, and high turnover [44-46]. In the BCT context, collaborative decision-making constitutes far more than relying on factual information, as patient communication may generate a decision that contradicts the evidence; the evidence may suggest a different plan. This highlights the reality that communication between the MDT and patient allows for the consideration of all factors [47].

Effective tools and processes, such as value-based breast cancer care, cannot be effectively addressed without interactions [48]. This does not neglect the role of tools and processes in the BCT context. Tools and processes should be used to improve the provision of patient-centered BCT planning and interactions [18]. The National Coalition for Cancer Survivorship (NCCS) provides the Cancer Survival Toolbox, a tool that aids cancer patients to learn how to communicate, obtain information, make decisions, negotiate, and speak up for their patient rights as they manage their cancer treatment [49].

When a person is diagnosed with breast cancer, it not only affects the individual but also has a significant impact on their family members. According to some studies, it is viable and beneficial to shift care delivery toward person- and family-centered care to clarify and address patient preferences, legitimize care-partner contributions, and grant appropriate access to information for family members and other care partners who are key players in enacting high-quality cancer care [50]. Research indicates that involving family members as care partners in communication intervention at the point of care increases patient understanding of illness, patient access to and use of the patient portal, and viewing of clinician visit notes among patients with more actively engaged care partners [50].

In conclusion, the agility of this BCT value fully conforms to the corresponding software engineering value. This leads us to classify this BCT value as achieving agility.

#### Value 2: Documentation and Quality of Documentation is as High Priority as Delivering an Appropriate Patient-Centered Treatment Plan

This software engineering agile value places “working software over comprehensive documentation.” This indicates that it is a higher priority to rapidly deliver functioning software than to spend great attention and effort on documentation tasks [18]. This does not neglect the importance of documentation. The aim is to improve future software releases through obtaining feedback quickly [18]. This value is translated into BCT language as follows: “Documentation and quality of documentation is as high priority as delivering an appropriate patient-centered treatment plan.”

Documentation is vital for medical records and legal purposes in parallel to the patient’s health and immediate needs. Patient-centered treatment planning’s main objective is to involve patients and their families in meaningful and in-depth conversations with their medical professionals to develop an accurate, well-thought-out treatment plan that appropriately uses all available medical information while also taking the medical, social, and cultural needs and preferences of the patient and family into account [26]. The treatment plan is designed, developed, communicated, and executed based on the patient’s individual characteristics, preferences, and responses to treatment in collaboration with an MDT that includes members with different specialties, thus ensuring that their best interests are considered and the best benefits are delivered. Therefore, all this necessary information should be documented as required by the MDT [26].

The documentation of a breast cancer patient may require up to 20 distinct professional groups. Across these different professions, most of this paperwork is normally completed by resident doctors [51]. Across different medical specialties, most of the documentation is carried out by gynecologists or gynecological staff. Most of the time dedicated to paperwork is required for therapy [51]. All this necessary information from different resources is required to be documented for organization, doctor-patient communication, quality assurance and management, managing future changes (as changes are inevitable during BCT), and, mostly, for diagnostic needs and follow-up after each therapy [51].

In a study carried out by the American Society of Clinical Oncology (ASCO), a treatment plan and summary template increased communication between patients and their health care providers among almost 90% of assessed patients and health care professionals [52]. This highlights the importance of documentation for communication and demonstrates not only the need to prioritize documentation but also the need to prioritize documentation quality requirements [51].

Thus, the agility of this BCT value does not conform with the corresponding agile value in software engineering.

#### Value 3: Patient Collaboration Over Treatment Plan Design Agreement

In software engineering, this agile value places “customer collaboration over contract negotiation.” It highlights that contracts do not clearly detail customer needs; rather, customers themselves do. Thus, continuous feedback loops are a priority in this agile value to ensure that the developed product is effective and useful as per the customer’s needs. This indicates that agile development is customer-centric. However, this does not neglect the role of contracts. This value can be translated into BCT language as follows: “patient collaboration over treatment plan design agreement.”

BCT professionals interpret patients’ collaboration based on continuous feedback from them. This is observed in patient collaboration and in visits that begin early in the BCT process and should happen frequently. This close collaboration culture with patients has been reported to lead to significant benefits for them, particularly when they are unable to make decisions [44]. Close collaboration helps oncologists and their teams ensure they are delivering an effective, useful treatment solution to patients. When they talk to patients and their families, they build feedback into the treatment cycle and reduce risks [28,29,41].

Encouraging patient collaboration can be achieved through providing them the care and information they need according to their stage of breast cancer, as each stage varies in the aspects of information and associated delivery of care [31,32]. What was effective at one stage may not produce similar effectiveness in another stage. Therefore, it is highly recommended to look for communication alternatives that meet needs and preferences at each stage [53]. Sharing a treatment plan and summary template enhances patients’ and their families’ collaboration [52].

In conclusion, this leads us to classify this BCT value as achieving agility.

#### Value 4: Responding to Change Over Following a Plan

The world is not static. Therefore, a workable plan should never be static. This software engineering agile value highlights that changes in software development happen due to changes in the market, customer preferences, priorities, project management conditions, and business needs [18]. Thus, the agile mindset encourages reviewing and readjusting the plan based on new, emerging information. Regarding the BCT context, this value can be maintained as it is without changing any of its wording.

Changes in a BCT plan are inevitable and can occur for several reasons. Changes involve but are not limited to diagnosis results, follow-up assessments, surgical outcomes, health conditions, tumor characteristics, treatment response, side effects and tolerance, structural changes, resources, policies, disease recurrence, drug resistance, monitoring assessments, new research, and others. All these alert us to the need for a personalized response to the changes as a priority over following a static plan. For example, changing the sequence of treatment, in terms of radiotherapy and surgery, could improve outcomes and reduce side effects in terms of treatment complications and address safety and technical feasibility [54]. Lifestyle changes that involve exercise, diet, smoking, and alcohol have empowered patients psychologically and reduced the risk of recurrence and death [55-57].

Breast cancer is a complex disease in which not all patients can benefit from the same treatment. Thus, it is essential to go beyond conventional BCTs [58]. Changes in BCT may require developing a novel treatment that could be described as personalized [58]. Changes to the treatment plan could happen if the aim has changed. Aims could include preventing cancer recurrence, slowing growth of cancer, and managing symptoms of incurable cancer [59]. Accordingly, changes entail allocating roles that are put into place to address the aim of BCT and provide referral to clinical trials that are regulated to effectively address the desired treatment aim with minimum side effects [60]. Furthermore, a change may require halting the treatment to recover from adverse side effects [60].

Managing BCT entails evaluating the patient’s response to treatment from the perspectives of surgery, imaging, and medical oncology. Adjuvant treatment is guided by an appropriate surgical and pathological assessment and follow-up care that concentrates on identifying recurring illness with the goal of enhancing long-term survival [61]. Whatever the change that requires a response, the BCT team will strive to improve the patient’s quality of life.

Magnetic resonance imaging (MRI) is used to accurately identify stages of breast cancer by assessing the size and extent of the tumor within the breast and evaluating if it has spread to nearby lymph nodes or other structures [62]. This information is essential in determining an appropriate treatment approach that is ready to respond to changes. A major change in the treatment plan may involve the need for surgery and additional treatment [62-64].

In BCT, personalized treatment is a means of responding to a change and delivering patient-centric medicine. Knowing the molecular characteristics of breast cancer subtypes is necessary to create a tailored therapy and diagnosis [65]. It is necessary to establish molecular profiles and metrics for tailoring the appropriate treatment and evaluating its benefits and risks. Dose ratios and regimens are likely to change according to an identified combination therapy [66]. BCT decisions are based not only on the assessment of prognostic factors but also on the assessment of pathological and clinical findings. A multitopic-based integrated data approach to address many breast cancer risk variables can bring tremendous insight and promises to change treatment for the better [67].

In conclusion, this BCT agile value shows how change is necessary for the benefit of the patient.

### Phase 5: Validating Breast Cancer Treatment Agile Values Through the Domain Expert

In this phase, the domain expert conducted a walkthrough of the output generated from the previous phase as a form of final validation. This was to ensure that the BCT agile values identified in phase 3 are consistent with the respective reported literature as shown in phase 4.

### Phase 6: Communicating and Disseminating Knowledge

The research knowledge obtained from this paper is disseminated in this open access journal to increase its availability and accessibility to the scientific community, policymakers, practitioners, and the public. In addition, the article is published to enhance collaboration between multidisciplinary fields for better BCT strategies. The authors also aim to deliver a public seminar about the ideas delivered in this article to increase community awareness of promoting agility in BCT for better care values, strategies, and delivery.

### Ethical Considerations

No ethical considerations are required for this study, as it does not involve human subjects. The statements for human subject research ethics review, exemptions, and approvals, as well as descriptions of informed consent (for the institutional review board), privacy and confidentiality protection, and compensation type and amount are not applicable. The study does not include any clinical setting for recruitment, not even recruitment procedures.

## Results

In this study, we have identified BCT agility values and showed how they conform to the concept of agility in the field of software engineering. Our validation was conducted through a comprehensive review of the existing literature and interviews with a breast cancer oncologist. This work has resulted in 4 BCT agility values, which are specified below:

1. Individuals and interactions over processes and tools (conforms)
2. Documentation and quality of documentation is as high priority as delivering an appropriate patient-centered treatment plan (does not conform)
3. Patient collaboration over treatment plan design agreement (conforms)
4. Responding to change over following a plan (conforms)

In summary, our analysis indicates that BCT has made significant strides in aligning with the concept of agility through the identification of the 4 values that were reconfigured from the software engineering field. These values focus on how individuals interact, collaborate, and welcome changes. However, the quality of documentation is as high priority as delivering a patient-centered treatment plan. These values have been integrated into various aspects of breast cancer care, from treatment planning and delivery to ongoing support and survivorship care. However, there is still room for improvement, and ongoing efforts to enhance these values in BCT will continue to benefit patients, families, BCT professionals, and health care providers.

## Discussion

The work has delivered a novel contribution to knowledge in identifying the first set of agile values in the BCT context adopted from the software engineering field. We conclude that only 3 of the 4 identified agile values in BCT conform, in terms of agility, with the 4 agile values in software engineering. The 3 BCT agility values are “individuals and interactions over processes and tools,” “patient collaboration over treatment plan design agreement,” and “responding to change over following a plan.” However, the second BCT agile value did not conform to agility, as it is vital that documentation not only be comprehensive but also have high quality and have high priority for delivering an appropriate patient-centered plan. None of the agile values were recorded as partially conforming to agility; this addresses the aim of this paper to identify BCT agile values and compare their conformance to agile values in the software engineering field.

In the first BCT agility value, the priority of collaboration has emerged as a fundamental aspect of BCT that is interpreted in the SDM and MDT approaches. MDTs consisting of oncologists, radiologists, surgeons, nurses, and other health care professionals work closely together to ensure comprehensive patient care. Furthermore, the involvement of patients in SDM has promoted collaboration between health care providers, patients, and those managing the treatment. Collaboration and ongoing interactions appear early in the BCT lifecycle and continue even after survival, emphasizing the significance of these interactions and implying that quality of life requirements are increasing and leading to more patient-centric processes. For the second BCT agility value, the design of BCT plans respects the personalized characteristics of each patient. All kinds of collaborations and interactions between the patient and the MDT to discuss and design an effective and safe BCT plan should be based on high-quality documentation. Therefore, it is a priority to allocate enough time to facilitate documentation work to attain agility in the remaining values. In the third agile value, patient collaboration is a higher priority than following a rigid treatment plan; this leads to better treatment decisions via continuous feedback throughout the BCT lifecycle. Regarding the fourth agile value, effective and safe changes are always welcomed to interrupt a BCT plan for the patient’s benefit, with or without minimizing impacts on quality of life, thereby leading to closer, patient-centric, and better treatment outcomes, as well as minimized side effects.

Attempts to address BCT agile values are limited and constrained by cultural mindsets, the availability of resources for implementation, the availability of quality standards that support agility, and interoperability of health care systems. Patients’ efficient participation is constrained by the quality of their education and their health literacy in terms of understanding risks, benefits, overall quality of life, and adherence to their treatment plans and medication regimens.

The authors do not claim that these are the only BCT agile values, as they are not limited to 4. Instead, the values proposed here constitute the first version of a BCT agile values manifesto. The implication of having BCT agile values shared among administration and BCT professionals is broader than the specific interpretation behind each identified BCT agile value. The identified agile values focus on effective and efficient collaboration between the patient and MDT in SDM, with consideration to continuous response to change, which will contribute to increasing the patient’s activity level and forming a more personalized BCT process that drives patient-centric therapy decisions. Moreover, this contributes to eliminating waste, in other words, unneeded activities or tasks that may involve tests, procedures, and treatments. Hence, this contributes to more cost-effective, time-saving, and efficient care. Rather than just following strict protocols, BCT agile values increase the flexibility of these protocols, allowing for more personalized care with respect to patients’ characteristics, preferences, and needs. The identified BCT agile values highlight the necessity of specifying quality assurance processes that aim at investigating the extent to which they conform to the concept of BCT agility. Health care systems are anticipated to become more flexible, patient-focused, and efficient by adopting agile values in BCT and care. This will eventually enhance patient outcomes and experiences and promote a change from inflexible, one-size-fits-all methods of care to ones that are tailored to the specific requirements of each patient and the rapidly advancing fields of medicine and technology. Finally, this holds the potential to revolutionize the way we approach oncological care. This endeavor reflects a commitment to embracing innovation, collaboration, and patient-centeredness, mirroring the very essence of the agile philosophy.

## Abbreviations

ASCO: American Society of Clinical Oncology
BCT: breast cancer treatment
MDT: multidisciplinary team
MRI: magnetic resonance imaging
NCCS: National Coalition for Cancer Survivorship
SDM: shared decision-making

## Data Availability

Data sharing is not applicable to this article as no data sets were generated or analyzed during this study.
